# Moral injury and substance use disorders among US combat veterans: results from the 2019–2020 National Health and Resilience in Veterans Study

**DOI:** 10.1017/S0033291721002919

**Published:** 2023-03

**Authors:** Shira Maguen, Brandon Nichter, Sonya B. Norman, Robert H. Pietrzak

**Affiliations:** 1 San Francisco VA Health Care System, San Francisco, CA, USA; 2 University of California – San Francisco, San Francisco, CA, USA; 3 National Center for PTSD, White River Junction, VT, USA; 4 Department of Psychiatry, University of California San Diego, La Jolla, CA, USA; 5 VA Center of Excellence for Stress and Mental Health, San Diego, CA, USA; 6 National Center for PTSD, VA Connecticut Healthcare System, West Haven, CT, USA; 7 Department of Psychiatry, Yale School of Medicine, New Haven, CT, USA; 8 Department of Social and Behavioral Sciences, Yale School of Public Health, New Haven, CT, USA

**Keywords:** Moral injury, alcohol use disorder, drug use disorder, substance use disorder, veteran, mental health

## Abstract

**Background:**

Exposure to potentially morally injurious events (PMIEs) is associated with increased risk for substance use disorders (SUDs), although population-based studies remain limited. The goal of this study was to better understand the relationships between PMIE exposure and lifetime and past-year alcohol use disorder (AUD), drug use disorder (DUD), and SUD.

**Methods:**

Data were analyzed from the 2019–2020 National Health and Resilience in Veterans Study, which surveyed a nationally representative sample of 1321 combat veterans. Multivariable analyses examined associations between three types of PMIE exposure (perpetration, witnessing, and betrayal), and lifetime and past-year AUD, DUD, and SUD, adjusting for sociodemographic variables, combat exposure severity, prior trauma, and lifetime posttraumatic stress disorder and major depressive disorder.

**Results:**

Perpetration was associated with increased odds of lifetime AUD (OR 1.15; 95% CI 1.01–1.31) and lifetime SUD (OR 1.18; 95% CI 1.03–1.35). Witnessing was associated with greater odds of past-year DUD (OR 1.20; 95% CI 1.04–1.38) and past-year SUD (OR 1.14; 95% CI 1.02–1.28). Betrayal was associated with past-year AUD (OR 1.20; 95% CI 1.03–1.39). A large proportion of the variance in past-year AUD was accounted for by betrayal (38.7%), while witnessing accounted for 25.8% of the variance in past-year DUD.

**Conclusions:**

Exposure to PMIEs may be a stronger contributor to SUDs among veterans than previously known. These findings highlight the importance of targeted assessment and treatment of moral injury among veterans with SUDs, as well as attending to specific types of morally injurious experiences when conceptualizing and planning care.

Moral injury has come to be recognized as a salient problem among veterans serving in war, with a burgeoning body of research proliferating over the last decade (Griffin et al., 2019). Moral injury is defined as a transgression of morals or values that are held personally or collectively, and may negatively affect emotional, functional (e.g. relationships, work), and spiritual domains (Litz et al., 2009). Research has also shown that there are a number of ways to classify potentially morally injurious events (PMIEs), including morally injurious events caused by one's own actions or inactions (e.g. perpetration), witnessing such events, or being betrayed by others (Bryan et al., 2016). A recent nationally representative sample of post-9/11 veterans surveyed shortly after leaving service found that a substantial proportion endorsed experiencing and being troubled by PMIEs, including acts of betrayal (41.1%), witnessing (27.9%), and perpetration (18.8%), demonstrating that these events are common among US veterans (Maguen et al., 2020*a*, *b*). Similarly, a prior all era population-based study of US combat veterans found slightly lower but significant rates of these PMIEs, with witnessing (25.5%) and betrayal (25.5%) being the most common (Wisco et al., 2017). These morally injurious events can lead to a ripple effect of consequences, most notably guilt and shame, as well as increasing risk for mental health disorders, suicidality, and other self-sabotaging behaviors (Griffin et al., 2019; Wisco et al., 2017).

There are now ample studies suggesting that moral injury is an independent construct that accounts for unique variance in outcomes, above and beyond the effects of posttraumatic stress disorder (PTSD), depression (MDD), and other mental health disorders (Bryan, Bryan, Roberge, Leifker, & Rozek, 2018; Griffin et al., 2019). Several studies have also found that exposure to PMIEs is associated with greater severity of PTSD and MDD symptoms, as well as heightened risk for suicidal thoughts and behaviors (Griffin et al., 2019; Maguen et al., 2020*a*, *b*; Nichter, Norman, Maguen, & Pietrzak, 2021; Wisco et al., 2017). Although researchers have speculated that moral injury may be linked to a greater likelihood of substance use disorders (SUDs; e.g. Capone et al., 2020), only four known studies have tested this relationship empirically, and to our knowledge, none have done so in a nationally representative veteran sample. In a study of 2797 US soldiers returning from Iraq, 40% of soldiers reported killing or being responsible for the death of another during their deployment (both forms of PMIEs). Even after controlling for combat exposure, killing and being responsible for the death of another was associated with elevated odds of alcohol abuse (Maguen et al., 2010). Similarly, a study of recently returning veterans from the Iraq war found that exposure to atrocities was associated with 61% increased odds of alcohol-related problems, even after adjusting for psychiatric comorbidities and combat exposure severity (Wilk et al., 2010). Among 1323 US Special Operations soldiers, fighting in combat was associated with alcohol misuse a few months post-deployment (Skipper, Forsten, Kim, Wilk, & Hoge, 2014). Finally, in a smaller community sample of veterans, exposure to morally injurious events was associated with alcohol misuse but not drug abuse (Kelley, Braitman, White, & Ehlke, 2019); however, this analysis did not adjust for other combat exposures or mental health symptoms. There are a few other studies that examine factors that mediate the relationship between exposure to PMIEs and substance use (e.g. Feingold, Zerach, & Levi-Belz, 2019) or examine relationships in the context of measurement validation (e.g. Nieuwsma et al., 2021). Thus, despite the fact that prior work has identified veterans with exposure to PMIEs as a high-risk group for SUDs (Maguen & Litz, 2012), no known studies have examined these associations in a nationally representative study of veterans.

Characterization of the association between exposure to PMIEs and SUDs, including both alcohol use disorder (AUD) and drug use disorder (DUD), is important for several reasons. According to the most widely used theoretical model of moral injury, guilt and shame are important hallmark symptoms of moral injury (Litz et al., 2009), and both of these distressing emotions are associated with substance use among veterans (Capone et al., 2020). More specifically, this model posits that PMIEs that result in guilt and other complex emotions might result in subsequent substance use, which may serve to avoid or numb these emotions. This model also suggests that self-sabotaging behaviors such as harmful alcohol or drug use may result from an inability to forgive and self-condemnation due to PMIEs (Litz et al., 2009). Furthermore, if veterans experience ongoing self-condemnation and self-loathing due to a failure to prevent acts that transgressed their own moral code, substance use can serve as a means of self-punishment, thus perpetuating the cycle of functional impairment. In the worst cases, this substance use can lead to overdose or suicidal behavior. Better understanding the nuanced relationship between exposure to PMIEs and substance use can therefore assist clinicians with providing more comprehensive evaluation and improved care to veterans and others with trauma histories.

In the current study, we provide the first known examination of the relation between exposure to PMIEs and SUDs in a nationally representative sample of US combat veterans. Given that most studies have typically examined more proximal relationships between exposure to PMIEs and SUDs, we sought to expand knowledge gaps by assessing relationships both within the past year, as well as lifetime histories of these disorders. This can provide a better sense of both the current and life course associations, given that these may differ. We employed additional analyses to help quantify the relative importance of each type of moral injury exposure (perpetration, witnessing, and betrayal) in relation to SUDs, while also considering other relevant risk factors for SUDs such as combat severity, prior trauma, and other common psychiatric morbidities (PTSD and MDD).

## Methods

Data were analyzed from the 2019–2020 National Health and Resilience in Veterans Study (NHRVS), which surveyed a nationally representative sample of 4069 US veterans. Inclusion criteria for the current study were that participants reported previous exposure to combat or a war zone, and 1321 veterans (weighted 35.0%) met this criterion. Sociodemographic and military characteristics of the sample are shown in Table 1.

**Table 1.** Sociodemographic, military, clinical, and MIES characteristics of US combat veterans

Variables	Weighted mean (s.d.) or *N* (weighted %)
*Sociodemographic characteristics*	
Age	59.1 (16.6)
Male sex	1270 (93.7%)
Caucasian race	1081 (75.1%)
College graduate or higher education	651 (37.1%)
Married or living with partner	996 (72.0%)
Currently employed	521 (50.0%)
Annual household income >$60 K	856 (63.9%)
*Military characteristics*	
Enlistment status	
Enlisted	996 (77.4%)
Commissioned	187 (11.9%)
Drafted	169 (10.7%)
Branch of service	
Army	543 (47.5%)
Navy	322 (22.3%)
Air Force	251 (14.6%)
Marine Corps	108 (6.3%)
National Guard, Reserves, Coast Guard	129 (9.3%)
Years of military service	
<4	366 (24.2%)
4–9	483 (38.9%)
10–19	113 (10.0%)
20 +	391 (26.9%)
War era	
World War II	20 (1.5%)
Korean War	56 (4.7%)
Persian Gulf War	235 (17.9%)
Vietnam War	789 (41.6%)
Iraq/Afghanistan War	264 (33.4%)
Other	111 (10.1%)
Number of deployments	
1	817 (59.6%)
2	254 (20.5%)
3+	246 (19.9%)
Year of last deployment (median, IQR)	1971 (1968, 1994)
Combat Exposure Scale score	10.4 (9.6)
Light exposure	747 (52.3%)
Light-moderate exposure	273 (19.8%)
Moderate exposure	194 (16.2%)
Moderate-heavy exposure	121 (10.3%)
Heavy exposure	18 (1.4%)
*Clinical characteristics*	
Number of lifetime traumas	11.2 (9.4)
Lifetime MDD	216 (18.3%)
Lifetime PTSD	189 (17.6%)
Lifetime SUD	599 (44.8%)
Lifetime AUD	563 (42.4%)
Lifetime DUD	148 (12.3%)
Current SUD	217 (19.1%)
Current AUD	144 (12.7%)
Current DUD	104 (9.3%)
*Moral Injury Events Scale characteristics*	
Scores	
MIES total score	2.0 (1.1)
Perpetration subscale	1.7 (1.1)
Witnessing subscale	2.5 (1.6)
Betrayal subscale	2.1 (1.3)
Prevalence of endorsement	Weighted % (s.e.)
Perpetration	11.2 (0.9)
Witnessing	23.1 (1.2)
Betrayal	24.5 (1.3)

s.d., standard deviation; s.e., standard error; MDD, major depressive disorder; PTSD, posttraumatic stress disorder; SUD, substance use disorder; AUD, alcohol use disorder; DUD, drug use disorder.

Raw frequencies are reported, while post-stratification weighting was applied to all other variables to permit generalizability to the US Veteran population.

Sum of percentages for war era does not equal 100 as overlapping endorsements were permitted.

Prevalence of endorsement of each category of PMIE was defined as responding ‘moderately agree’ or ‘strongly agree’ for any item on the corresponding subscale.

The MIES total score and subscales were calculated as the mean across items to permit comparisons across subscales. Each MIES subscale had a possible range of 1–6.

The study rationale and sampling methodology of the NHRVS have been described previously (Tsai, Schick, Hernandez, & Pietrzak, 2020). Veterans completed a 50 minute, anonymous, web-based survey. The NHRVS sample was drawn from KnowledgePanel^®^, a research panel of more than 50 000 households maintained by Ipsos, Inc. KnowledgePanel^®^ is a probability-based, online, non-volunteer access survey panel of a nationally representative sample of US adults that covers approximately 98% of US households. Panel members were recruited through national random samples, originally by telephone and now almost entirely by postal mail. KnowledgePanel^®^ recruitment uses dual sampling frames that include both listed/unlisted telephone numbers, telephone/non-telephone households, and cell-phone-only households, as well as households with and without Internet access. To allow for generalizability of study results to the entire population of US veterans, Ipsos statisticians computed post-stratification weights using the following benchmark distributions of US military veterans from the most recent (August 2019) Current Veteran Population Supplemental Survey of the US Census Bureau's American Community Survey: age, gender, race/ethnicity, Census Region, metropolitan status, education, household income, branch of service, and years of military service. An iterative proportional fitting (raking) procedure was used to produce the post-stratification weights. All participants provided informed consent and the Human Subjects Committee of the VA Connecticut Healthcare System approved the study. The authors assert that all procedures contributing to this work comply with the ethical standards of the relevant national and institutional guides on human research.

### Assessments

#### Sociodemographics

We adjusted for the following demographics: age (continuous), gender (male, female), marital status (married or partnered *v.* not), education (college graduate or more, some college or less), annual household income (<$60 000, >$60 000).

#### Potentially morally injurious experiences (PMIEs)

Exposure to PMIEs was assessed using The Moral Injury Events Scale (MIES; Nash et al., 2013), a nine-item self-report measure. Ratings are on a six-point Likert scale, ranging from 1 (strongly disagree) to 6 (strongly agree). The MIES total score (Cronbach's *α* = 0.90) and perpetration, witnessing, and betrayal subscales showed acceptable-to-excellent internal consistency in this sample (*α*'s = 0.91, 0.85, 0.78, respectively). Prevalence of endorsement of each category of PMIE on the MIES was defined as responding ‘moderately agree’ or ‘strongly agree’ for any item on the corresponding subscale (parallel to Wisco et al., 2017). We then averaged scores for each of the items per subscale to yield a mean total score for perpetration, witnessing, and betrayal subscales.

#### Posttraumatic stress disorder (PTSD)

Lifetime probable PTSD was assessed using a modified version of the Posttraumatic Stress Disorder Checklist for DSM-5 (Weathers et al., 2013) that assessed lifetime PTSD symptoms (*α* = 0.96); a cut-off score of ⩾33 was indicative of a positive screen for PTSD (Bovin et al., 2016). PTSD symptoms were assessed in relation to veterans' worst traumatic event endorsed on the Life Events Checklist for DSM-5 (Weathers et al., 2013).

#### Major depressive disorder (MDD)

Lifetime probable MDD was assessed using a modified self-report version of the Major Depressive Disorder module from the DSM-5 version of the Mini Neuropsychiatric Interview (Sheehan, 2016).

#### Alcohol use disorder (AUD)

Lifetime probable AUD was assessed using a modified self-report version of the Alcohol Use Disorder module from the DSM-5 version of the Mini Neuropsychiatric Interview (Sheehan, 2016). Past-year probable AUD was assessed using the Alcohol Use Disorders Identification Test (AUDIT; Saunders, Aasland, Babor, De La Fuente, & Grant, 1993); a cut-off score of ⩾8 was indicative of a positive screen for current AUD; *α* = 0.85.

#### Drug use disorder (DUD)

Lifetime probable DUD was assessed using a modified self-report version of the Drug Use Disorder module from the DSM-5 version of the Mini Neuropsychiatric Interview (Sheehan, 2016). Past-year DUD was assessed using the Screen of Drug Use (SDU; Tiet et al., 2015); a response of ⩾7 days to the question ‘How many days in the past year have you used non-prescription drugs?’ or if the response to this question is 6 or fewer days, a response of ⩾2 days to the question ‘How many days in the past 12 months have you used drugs more than you meant to?’ is indicative of a positive screen for past-year DUD.

#### Substance use disorder (SUD)

Lifetime probable SUD was operationally defined in the current study as meeting the criteria for lifetime AUD and/or DUD on the Mini Neuropsychiatric Interview (Sheehan, 2016). Veterans met the criteria for past-year probable SUD if they screened positive for probable AUD and/or DUD on the AUDIT and SDU.

#### Combat exposure

Lifetime combat exposure was assessed using the Combat Exposure Scale (CES; Keane et al., 1989), which measures the frequency of exposure to seven types of combat experiences (e.g. number of times under enemy fire, going on combat patrols or other dangerous duty); *α* = 0.86. Scores are categorized to reflect the intensity of combat: 0–8 = light; 9–16 = light-moderate; 17–24 = moderate; 25–32 = moderate-heavy; and 33–41 = heavy.

#### Trauma exposure

Lifetime trauma exposure was assessed using the Life Events Checklist for DSM-5 (Weathers et al., 2013), which is a self-report measure that assesses for the lifetime occurrence of 16 potentially traumatic events and one ‘other’ event, and whether the event ‘happened to me’, ‘witnessed it happen to someone else’, ‘learned about it happening to close family member or friend’, and/or ‘exposed to it as part of my job’ (e.g. paramedic, police officer, military, or other first responder). Endorsement of potentially traumatic events was summed (range = 0–68).

### Data analysis

Raw unweighted frequencies are reported and poststratification weights were applied when computing prevalence and inferential statistics to allow for generalizability to the US veteran population. Missing data (<5%) were imputed using chained equations.

Statistical analyses were conducted using SPSS version 27 statistical software. Analyses were performed in three steps. First, we computed descriptive statistics for the sample to summarize sociodemographic, military, clinical, and MIES characteristics. Second, we examined the association between exposure to three types of PMIEs (perpetration, witnessing, and betrayal subscales of the MIES) with lifetime and past-year AUD, DUD, and SUD in a series of multivariable logistic regression analyses; consistent with prior work (Nichter et al., 2021; Wisco et al., 2017), models adjusted for sociodemographic characteristics, combat exposure severity, lifetime trauma exposure, and lifetime PTSD and MDD. Prior to regression analyses, we conducted independent-samples *t* tests to compare MIES subscale scores in each of the dependent variable groups (i.e. past-year AUD, DUD, SUD; lifetime AUD, DUD, SUD). MIES subscale scores that differed significantly between groups at the *p* < 0.05 level were entered into multivariable logistic regression analyses. Third, to determine the relative contribution of each variable to the model explained variance (*R*^2^), we conducted relative importance analyses (Tonidandel & LeBreton, 2011) using the R statistical software package (R Core Team, 2020). This analysis decomposes the total variance explained in regression models into proportional contributions, while taking into consideration intercorrelations among independent variables, thus quantifying the relative importance of these variables in predicting outcomes.

## Results

Table 1 presents sociodemographic, military, trauma, and MIES characteristics of the sample. Approximately one-fourth of veterans endorsed at least one betrayal-based PMIE (24.5%) or witnessing PMIE item (23.1%), while 11.2% endorsed at least one perpetration-based item.

Bivariate analyses revealed that MIES scores were significantly higher in veterans who screened positive for all dependent variables (all *p*'s < 0.05), with the exception of betrayal scores, which did not differ between veterans with and without past-year DUD (*p* = 0.95). Table 2 displays the results of multivariable regression analyses examining the associations of MIES subscales with past-year and lifetime AUD, DUD, and SUD, adjusting for sociodemographic, military, and psychiatric variables. Results revealed that perpetration scores were independently associated with increased odds of lifetime AUD [odds ratio (OR) 1.15; 95% confidence interval (CI) 1.01–1.31] and lifetime SUD (OR 1.18; 95% CI 1.03–1.35). Witnessing scores were independently associated with greater odds of past-year DUD (OR 1.20; 95% CI 1.04–1.38) and past-year SUD (OR 1.14; 95% CI 1.02–1.28). Betrayal scores were independently associated with past-year AUD (OR 1.20; 95% CI 1.03–1.39).

**Table 2.** Multivariable associations between exposure to potentially morally injurious events and substance use disorders among US combat veterans

	Past-year AUD^a^	Past-year DUD^a^	Past-year SUD^a^	Lifetime AUD^a^	Lifetime DUD^a^	Lifetime SUD^a^
	OR (95%CI)	OR (95%CI)	OR (95%CI)	OR (95%CI)	OR (95%CI)	OR (95%CI)
Combat severity	1.02 (1.01–1.04)*	0.98 (0.95–1.00)	1.00 (0.99–1.02)	1.01 (0.99–1.02)	0.98 (0.96–1.00)	1.00 (0.99–1.01)
Cumulative trauma^b^	0.99 (0.97–1.01)	1.01 (0.99–1.03)	1.00 (0.98–1.02)	1.04 (1.03–1.05)***	1.02 (1.01–1.04)**	1.04 (1.02–1.05)***
Lifetime PTSD	1.75 (1.11–2.77)*	0.90 (0.51–1.60)	1.63 (1.08–2.45)*	0.75 (0.51–1.09)	0.71 (0.44–1.16)	0.79 (0.54–1.15)
Lifetime MDD	0.94 (0.83–1.16)	2.21 (1.31–3.70)**	1.01 (0.68–1.52)	3.50 (2.43–5.03)***	2.62 (1.70–4.05)***	3.74 (2.59–5.40)***
Perpetration	0.98 (0.83–1.16)	0.90 (0.74–1.09)	0.95 (0.82–1.11)	1.15 (1.01–1.31)*	1.12 (0.95–1.33)	1.18 (1.03–1.35)*
Witnessing	1.01 (0.88–1.16)	1.20 (1.04–1.38)*	1.14 (1.02–1.28)*	0.97 (0.87–1.07)	1.09 (0.95–1.26)	0.96 (0.87–1.07)
Betrayal	1.20 (1.03–1.39)*	–	1.07 (0.93–1.22)	0.92 (0.82–1.03)	1.12 (0.96–1.32)	0.96 (0.85–1.08)

OR, odds ratio; 95% CI, 95% confidence interval; AUD, alcohol use disorder; DUD, drug use disorder; SUD, substance use disorder; PTSD, posttraumatic stress disorder; MDD, major depressive disorder.

a All models were adjusted for sociodemographic covariates, including gender, age, race, education, income, and employment, and models shown are the fully adjusted models that also include combat severity, cumulative trauma, mental health variables, and PMIEs. ORs for MIES (perpetration, witnessing, and betrayal) represent the odds per unit change in the subscale score.

b Cumulative trauma refers to the total number of traumatic events endorsed on the Life Events Checklist for DSM-5. The analysis of past-year DUD did not include Betrayal scores because they were not associated with this outcome in a bivariate analysis (*p* = 0.95).

*p < .05, **p < .01, ***p < .001.

Results of a relative importance analysis revealed that the majority of the explained variance in lifetime AUD was accounted for by lifetime MDD (48.3%), greater lifetime trauma exposure (32.3%), younger age (6.3%), and perpetration (5.3%). The majority of the explained variance in past-year AUD was accounted for by younger age (42.6%), betrayal (38.7%), lifetime PTSD (16.2%), and combat exposure severity (2.5%).

The majority of the explained variance in lifetime DUD was accounted for by lifetime MDD (54.0%), younger age (20.3%), greater lifetime trauma exposure (13.8%), and lower educational attainment (8.7%). The majority of the explained variance in past-year DUD was accounted for by witnessing (25.8%), lifetime MDD (25.7%), younger age (22.5%), and lower household income (17.9%), with lower educational attainment and male gender explaining the remaining 6.3% and 1.8% of the variance in this outcome, respectively.

The majority of the explained variance in lifetime SUD was accounted for by lifetime MDD (51.4%), greater lifetime trauma exposure (27.8%), perpetration (7.9%), and lower household income (4.1%). The majority of the explained variance in past-year SUD was accounted for by younger age (48.8%), lifetime PTSD (31.6%), and witnessing (12.4%).

## Conclusions

To our knowledge, this study is the first to examine the relationships between exposure to PMIEs and SUDs, including both AUD and DUD, in a nationally representative sample of US combat veterans. We found that distinct forms of PMIEs were differentially associated with lifetime and past-year SUDs. Specifically, exposure to PMIEs involving transgressing or violating one's own core moral boundaries was associated with increased odds of lifetime AUD and SUD, while exposure to PMIEs characterized by witnessing others' moral transgressions was associated with greater odds of past-year DUD and SUD. Importantly, given that the current study sample was comprised predominantly of older combat veterans (mean age = 59), these findings suggest that exposure to PMIEs may influence both the development and current prevalence of SUD over several decades in this population. For example, results suggest that while perpetration-based PMIEs may be more strongly associated with ever-developing AUD and SUD following exposure to PMIEs and that morally injurious events characterized by witnessing others' immoral acts may be associated with current DUD or SUD. A significant limitation of prior literature on the impact of morally injurious experiences is that the majority of studies have examined associations between exposure to PMIEs and current mental health symptoms. To this end, the current study extends prior research by investigating both current and lifetime diagnoses, shedding light on the fact that the impact of exposure to PMIEs and associated mental health manifestations may need to be considered at various time points in order to more comprehensively understand these relationships.

It is also important to note that the current study found that exposure to PMIEs caused by perpetration was predominantly associated with AUD, while exposure to PMIEs by witnessing was more predominantly associated with DUD. The few existing previous studies that have examined associations between exposure to PMIEs and substance use have almost exclusively focused on AUDs, so this finding adds an important level of nuance that may have treatment implications. An important next step would be to examine the mechanisms of action or potential mediators of these associations. For example, one conceivable explanation is that guilt and/or shame may mediate the relationship between PMIEs related to the self and AUD, while remorse or helplessness may be more salient pathways between witnessing PMIEs and DUD. If such differential pathways were determined, different interventions may be important to examine in the context of treating veterans with moral injury and substance use problems. These are important directions for further research, especially given the burgeoning state of research on moral injury and substance use.

To evaluate the robustness of the observed associations between exposure to PMIEs and SUDs, we also examined the relative importance of each form of PMIE exposure in accounting for the variance in each SUD outcome. We found that over 5% of the variance in *lifetime* AUD (and 8% in lifetime SUD) was explained by exposure to PMIEs due to perpetration, following lifetime MDD and lifetime trauma exposure. Strikingly, nearly 39% of the variance in *current* AUD was accounted for by betrayal, which explained more variance than the contributions of MDD or PTSD. Betrayal has been linked to alcohol use in a prior study of civilians through pathways of difficulty discerning or disregarding risk and self-destructiveness, including self-harm and suicidality (Delker & Freyd, 2014); further research is needed to understand whether these pathways may be applicable to combat veterans exposed to PMIEs. Further, while MDD, PTSD, and prior trauma exposure are commonly considered when providing treatment for AUD, moral injury is typically not assessed or addressed as part of treatment, and there is currently no framework to help guide the treatment of AUD in veterans who suffer from moral injury. Collectively, these findings highlight the importance of moral injury in prevention and treatment efforts for AUD. Not taking exposure to PMIEs into account may delay recovery or exacerbate symptoms. Future studies should examine how to best incorporate the assessment and treatment of moral injury into substance use treatment.

Approximately 26% of the total explained variance in current DUD was accounted for by exposure to witnessing a morally injurious event (also over 12% of any SUD in the last year), explaining equally as much of the variance in this outcome as MDD. Given that exposures to PMIEs are not routinely assessed as part of screening for SUDs, they may be overlooked by clinicians who treat veterans with addictive disorders. If exposure to PMIEs contributes to veterans' substance use but is not addressed in treatment, it may be a reason why some relapse or do not have fully successful treatment outcomes. Indeed, our finding that witnessing a morally injurious event explained substantial variance in both past-year DUD and SUD suggests that exposure to PMIEs may be a more robust contributor to the etiology of SUDs in veterans than previously conceptualized.

Several potential explanations may underlie the observed associations between exposure to witnessing-based PMIEs and DUD. Witnessing a PMIE is often accompanied by a sense of powerlessness if veterans are not able to intervene to stop the incident, as well as a deep sense of regret in the aftermath. Consequently, veterans may question if they could have done more to stop or prevent the event, even if this was not feasible. Guilt and shame may also follow exposure to PMIEs, especially as more time passes. In concert, these complex reactions may create a foundation for coping through drug use in an attempt to numb emotions or memories of the PMIEs (Norman, Wilkins, Myers, & Allard, 2014). It may lead some to believe they do not deserve to get better, which may contribute to low rates of engagement in substance use treatment among symptomatic veterans (Goldberg et al., 2019). Researchers have not yet explored relationships between exposure to PMIEs and DUDs, likely due to the challenges of assessing for these issues, stigma, as well as ramifications of revealing drug use problems while military personnel are still in the service.

Taken together, these findings underscore the importance for clinicians to assess for exposure to PMIEs when working with veterans with or at risk for SUDs. Conversely, it may also be important to assess for alcohol and drug use when veterans present with a history of exposure to morally distressing experiences. Research supporting various treatments for moral injury has been accumulating over the last few years, and treatments such as Trauma-Informed Guilt Reduction Therapy (Norman et al., 2014), Impact of Killing (Maguen et al., 2017), Adaptive Disclosure (Gray et al., 2012), Acceptance and Commitment Therapy for Moral Injury (Nieuwsma et al., 2015), and Building Spiritual Strength (Harris et al., 2011) may be considered. However, it is important to note that most of the treatment trials for these modalities have excluded individuals with SUDs or only included those with mild SUDs. Consequently, mixed-method research is needed to better understand at which phase of treatment it would be helpful for individuals with SUDs to receive these treatments, as well as mechanisms through which individuals with a history of or heightened risk for the development of SUDs might benefit. Importantly, moral injury and substance use also adversely impact spouses, children, and families, so future research should also consider the broader family unit when designing and evaluating the efficacy of interventions.

Several limitations of this study should be noted. Although a strength of this study is that it utilized a nationally representative sample of US combat veterans, future work may want to oversample veterans of recent war eras, given that younger age was a consistent variable that emerged when explaining the variance in current and lifetime SUD outcomes. Relatedly, while moral injury has most frequently been studied in the context of war, research is beginning to expand to other populations, such as healthcare workers and first responders who have to make life or death decisions in the context of their work (Griffin et al., 2019). Future research would help expand knowledge regarding the relation between moral injury and SUDs in these populations. Another notable finding that emerged in the current study was that the impact of morally injurious events varied by outcome and assessment period (past year *v.* lifetime), so future longitudinal studies in younger veterans and other populations exposed to PMIEs could help shed light on the impact of exposure to PMIEs at various developmental periods. The current study was also cross-sectional, so temporality cannot be ascertained from the observed associations (e.g. whether lifetime SUDs pre-existed prior to exposure to PMIEs). Finally, we were unable to examine mediators (e.g. guilt/shame) between exposure to PMIEs and SUDs in the current study, and future longitudinal research examining the potential mechanistic links between these variables is needed to guide treatment efforts. For example, interpersonal support may be an important mediator to consider in this context. Other variables to consider include history of prior mental health treatment and physical pain, which may be associated with SUDs.

Notwithstanding these limitations, the current study is the first, to our knowledge, to examine the population-based burden of exposure to PMIEs on SUDs in a nationally representative sample of US combat veterans. Results revealed that perpetration-based PMIEs were strongly associated with lifetime AUD and SUD and that witnessing-based PMIEs were most robustly linked with current DUD and SUD. These findings highlight the importance of assessment and treatment of moral injury-based traumas among veterans with SUDs, as well as future research that focuses on the treatment of concurrent moral injury and SUDs.
